# Insecticides Promote Inflammation and Gut Barrier Alteration in In-Vitro Human Models

**DOI:** 10.3390/jox16020066

**Published:** 2026-04-13

**Authors:** Carlos Sanchez-Martin, Mariagrazia D’Agostino, Stefano Miglietta, Veronica Cocetta, Luna Laera, Isabella Giacomini, Martina Lanza, Marica Mennini, Maria Maddalena Storelli, Ettore Cicinelli, Monica Montopoli, Alessandra Castegna

**Affiliations:** 1 Department of Experimental Medicine, University of Rome Tor Vergata, Via Montpellier 1, 00133 Rome, Italy; carlos.sanchez.martin@uniroma2.it; 2 Department of Biosciences, Biotechnologies and Environment, University of Bari Aldo Moro, Via Orabona 4, 70125 Bari, Italy; mariagrazia.dagostino@uniba.it (M.D.); stefano.miglietta@uniba.it (S.M.); luna.laera@uniba.it (L.L.); m.mennini@studenti.uniba.it (M.M.); mariamaddalena.storelli@uniba.it (M.M.S.); 3 Department of Pharmaceutical and Pharmacological Sciences, University of Padova, Largo Meneghetti 2, 35131 Padova, Italy; veronica.cocetta@unipd.it (V.C.); isabella.giacomini@unipd.it (I.G.); 4 Department of Experimental Medicine, University of Salento, Via per Monteroni, 73100 Lecce, Italy; martina.lanza@unisalento.it; 5 Second Unit of Obstetrics and Gynecology, Department of Biomedical and Human Oncological Science, Università degli Studi di Bari Aldo Moro, Piazza Giulio Cesare 1, 70124 Bari, Italy; ettore.cicinelli@uniba.it

**Keywords:** insecticides, inflammation, pro-inflammatory macrophages, barrier integrity, tight junction organization

## Abstract

Background: The extensive use of insecticides in modern agriculture has raised concerns about potential chronic effects on human health beyond acute toxicity. Limited evidence exists regarding their impact on immune regulation and intestinal barrier integrity, two key components of host-environment interactions. Methods: Human in-vitro models were used to investigate the immunomodulatory and intestinal effects of several commonly used agricultural insecticides. Primary human macrophages derived from peripheral blood mononuclear cells were exposed to insecticides to assess cell viability and polarization status. Intestinal barrier function was evaluated using Caco-2 cell monolayers by measuring oxidative stress, epithelial integrity, paracellular permeability, and tight junction organization. Results: The tested insecticides induced a pro-inflammatory macrophage phenotype, characterized by increased expression of M1 markers and reduced M2 markers, without affecting cell viability. In Caco-2 cells, insecticide exposure compromised epithelial barrier integrity and disrupted tight junction organization. In this context, neither Spinetoram nor Spirotetramat induced notable oxidative stress under pro-oxidant conditions. However, Spirotetramat caused a significant increase in paracellular permeability. Conclusions: These findings indicate that commonly used insecticides can modulate immune responses and impair intestinal barrier function, suggesting potential mechanisms by which chronic low-level exposure may contribute to immune dysregulation and epithelial dysfunction in humans.

## 1. Introduction

Rapid population growth and increasing pressure on global food supplies have driven the intensification and expansion of modern agricultural systems [1]. Within this scenario, insecticides have become indispensable tools for controlling and eradicating pest insects, enhancing productivity and ensuring effective crop protection [2,3]. These compounds are primarily classified as carbamates (e.g., Formetanate) which inhibit acetylcholinesterase at neuronal synapses [2], and neonicotinoids (e.g., Acetamiprid) that bind to nicotinic acetylcholine receptors [4], causing continuous nerve stimulation, hyperexcitation, and ultimately paralysis and insect death. Other insecticides act by disrupting insect metabolism [5], thereby interfering with growth and development (e.g., Spirotetramat) or by inducing intracellular calcium release, leading to paralysis and cessation of feeding (e.g., Chlorantraniliprole) [6]. Compared with alternative pest management strategies, insecticides offer several advantages, including ease of application, reliability, and high efficacy against specific target organisms. These factors have contributed to their widespread and increasing demand [7]. A broad variety of insecticides is also extensively used to prevent vector-borne plant disease that negatively affect agriculture and natural ecosystems, leading to reduced crop yields, economic losses and ecological disturbances [8,9]. Nevertheless, the increased and prolonged utilization of these chemicals in agriculture over the past few decades has led to unintended consequences for soil and water resources, as well as adverse effects on non-target organisms. Such impacts can disrupt ecosystems, pollute groundwater, and threaten agricultural sustainability, animal welfare, and human health [10]. Although many insecticides are rapidly degraded or metabolized after application, high concentrations or prolonged exposure can be highly detrimental, as these compounds may accumulate and transfer along the food chain [2]. Beyond environmental damage, insecticide overuse poses significant risks to human health, with reported effects ranging from nausea, headaches, and skin irritation to more severe outcomes such as carcinogenesis and renal impairment [3]. Depending on exposure levels and duration, insecticides may also affect the central nervous system, muscle function, respiratory physiology, reproductive capacity, and endocrine homeostasis [11]. Consequently, regulatory authorities, including the World Health Organization (WHO), have implemented strict laws and safety guidelines to reduce harmful effects and safeguard public health [12].

Macrophages are central components of the innate immune system, capable of sensing diverse signals and triggering distinct activation responses that can be recapitulated in-vitro by pro-inflammatory M1 and anti-inflammatory M2 phenotypes [13]. Pro-inflammatory macrophages play a pivotal role in host defense by eliminating pathogens and initiating inflammatory responses, whereas M2 macrophages exhibit an anti-inflammatory transcriptional signature that supports the resolution of inflammation, tissue repair, and regeneration. Furthermore, dysregulated activation of pro-inflammatory macrophages has been associated with the loss of autoimmune tolerance, contributing to the development of autoimmune disorders [14]. Although several studies have shown that exposure to insecticides can elicit inflammatory responses in both humans and animal models [15,16,17], their specific impact on macrophage function remains largely unexplored.

Based on these findings, we investigated the immunomodulatory effects of a panel of insecticides on human macrophages derived from peripheral blood mononuclear cells (PBMCs) as well as on in-vitro models of the human gastrointestinal barrier. Our findings contribute to a clearer understanding of how insecticides influence macrophage polarization and may help to better define the potential risks associated with prolonged exposure to these compounds.

## 2. Materials and Methods

### 2.1. Chemicals and Stock Solutions

Phosphate-buffered saline (PBS), RPMI 1640 medium, fetal bovine serum (FBS), L-glutamine, penicillin, and streptomycin were purchased from Thermo Fisher Scientific (Waltham, MA, USA), while high-glucose Dulbecco’s Modified Eagle Medium (DMEM) and Hanks’ Balanced Salt Solution were obtained from Corning Inc. (Corning, NY, USA). Ethylenediaminetetraacetic acid (EDTA), bovine serum albumin (BSA), dimethyl sulfoxide (DMSO), and protease inhibitor cocktails were sourced from Sigma-Aldrich (Darmstadt, Germany). Lymphoprep™ density gradient medium was supplied by STEMCELL Technologies (Vancouver, BC, Canada). Human granulocyte-macrophage colony-stimulating factor (GM-CSF), macrophage colony-stimulating factor (M-CSF), lipopolysaccharide (LPS), interferon gamma (IFNγ), and interleukin-10 (IL-10) were acquired from ImmunoTools (Friesoythe, Germany). The insecticides Formetanate, Acetamiprid, Spirotetramat, Spinetoram, and Chlorantraniliprole were provided by ReAgri S.r.l (Massafra, Italy). Hoechst dye, anti-Occludin primary antibody, Fluoromount-G™ Mounting Medium, fluorescein isothiocyanate (FITC) and 2′,7′-dichlorofluorescin-diacetate (H_2_-DCF-DA) were obtained from Invitrogen (Carlsbad, CA, USA) and Sigma-Aldrich.

### 2.2. Cell Culture

Human monocytes were isolated from buffy coats obtained from three different healthy blood donors (*n* = 3), collected on different dates under an institutional review board-approved protocol (0030759, 28 March 2023), using CD14-Microbeads as previously described [18,19]. Briefly, buffy coats were diluted in Ca^2+^/Mg^2+^-free PBS containing 1 mM EDTA and loaded into SepMate™ tubes with Lymphoprep™ density gradient medium to isolate PBMCs. The opaque interphase containing PBMCs was collected, washed, and centrifuged again to eliminate residual debris. The PBMC pellet was resuspended in cold PBS supplemented with 2 mM EDTA and 0.5% bovine serum albumin (BSA) and incubated with anti-CD14 magnetic microbeads. After 15 min of incubation, CD14^+^ monocytes were centrifuged and purified using LS columns. Monocytes were cultured in RPMI 1640 supplemented with 10% FBS, 2 mM L-glutamine, and 1% penicillin and streptomycin, and differentiated into macrophages for six days in the presence of GM-CSF or M-CSF, depending on the desired macrophage phenotype. On day seven, macrophages were pretreated with insecticides for 1 h and subsequently polarized for 24 h using either 100 ng/mL LPS combined with 20 ng/mL IFNγ for M1 polarization, or 10 ng/mL IL-10 for M2 polarization. Insecticides were dissolved in DMSO and used at concentrations ranging from 0.05 to 50 µM, while control samples received the vehicle alone. Tested insecticide concentrations are within the typical residue ranges observed in fruits and vegetables and comply with the European Union’s maximum residue limits (MRLs) according to the EU Pesticides Database (https://food.ec.europa.eu/plants/pesticides/eu-pesticides-database_en, accessed on 12 March 2026).

Caco-2 cell line (RRID: CVCL_0025) was purchased from ATCC (Manassas, VA, USA) and used as a well-established in-vitro model of the intestinal epithelial barrier. Cells were cultured in DMEM, supplemented with 10% FBS, 2 mM L-glutamine, 100 IU/mL penicillin, and 100 µg/mL streptomycin, and maintained at 37 °C in a humidified atmosphere with 5% CO_2_.

### 2.3. Cell Viability Assays

Macrophage viability following insecticide exposure was assessed using the Alamar Blue assay (Bio-Rad Laboratories Inc., Hercules, CA, USA). Macrophages were pretreated with insecticides for 1 h and subsequently polarized with LPS and IFNγ for 24 h to induce an M1-like phenotype. The culture medium was then replaced with 100 µL of fresh RPMI 1640 medium containing 10% Alamar Blue reagent before incubating cells at 37 °C for 5 h. Absorbance was recorded at 570 and 600 nm using a FLUOstar^®^ Omega microplate reader (BMG Labtech, Ortenberg, Germany).

Caco-2 cells were plated in 96-wells plates and treated with insecticides according to the experimental protocol. Cells were then fixed with 4% paraformaldehyde (PFA), stained with Crystal Violet and solubilized with 1% acetic acid. Cell viability was quantified by measuring the absorbance at 570 nm with a VICTOR™ X3 2030 multilabel plate reader (PerkinElmer Inc., Waltham, MA, USA). Caco-2 cell proliferation was evaluated following treatments with 50 μM of Spinetoram or Spirotetramat, alongside the untreated control condition. After 24 h, cells were harvested, stained with Trypan Blue, and viable cells were counted using Countess 3 Automated Cell Counter (Thermo Fisher Scientific, Waltham, MA, USA). Cell counts at 24 h were normalized to the initial cell number (0 h) and expressed as % proliferation rate.

### 2.4. qRT-PCR of Inflammatory Markers

RNA extraction, cDNA synthesis, and quantitative real-time PCR (qRT-PCR) analysis were performed according to protocols described in previous studies [20,21,22]. TaqMan probes targeting human *CXCL10*, *CD80*, *TNF*, *CD86*, *CD163* and *CD206* were obtained from Integrated DNA Technologies Inc. (Coralville, IA, USA), while the TaqMan assay for human *ACTB* (Thermo Fisher Scientific) was used as the internal reference gene. Relative gene expression levels were calculated using the 2^−ΔΔCt^ method and expressed as fold change relative to control samples.

### 2.5. Reactive Oxygen Species Measurement

Intracellular reactive oxygen species (ROS) levels were measured using H_2_-DCF-DA probe, as previously described [23]. After cellular uptake, the probe H_2_-DCF-DA was deacetylated by intracellular esterases and subsequently oxidized to the fluorescent compound 2′,7′-dichlorofluorescein (DCF). Caco-2 cells were seeded in 96-wells black plates and exposed for 24 h to increasing concentrations of Spinetoram and Spirotetramat. After treatment, cells were incubated with H_2_-DCF-DA probe at 100 μM for 30 min at 37 °C. Basal fluorescence was measured using a VICTOR™ X3 multilabel plate reader (PerkinElmer) at an excitation wavelength of 485 nm and an emission wavelength of 535 nm. Following the initial baseline measurement, H_2_O_2_ was added directly to the wells to induce acute oxidative stress, and fluorescence was immediately recorded.

### 2.6. Transepithelial Electrical Resistance Assay

Caco-2 cells were plated at the desired density on transparent polyester Transwell™ inserts (0.45 μm pore size, 1 cm^2^ surface area; BD Falcon™, Corning, NY, USA) placed in 24-wells plates and cultured under standard conditions. The medium was changed every two days until the formation of a fully differentiated, confluent epithelial monolayer. Barrier integrity was monitored by measuring transepithelial electrical resistance (TEER) between days 14 and 21 after seeding using a Millicell^®^ ERS volt–ohm meter (EMD Millipore Corporation, Billerica, MA, USA) with chopstick electrodes [24]. After TEER stabilization, Spinetoram and Spirotetramat, dissolved in Hanks’ Balanced Salt Solution (HBSS), were applied to the apical chamber at concentrations of 0.05, 0.5, 5, and 50 μM, while the basolateral chamber received HBSS alone. TEER values were recorded at 0, 3, 6, 21, and 24 h of treatment, following an equilibration period of 30 min at room temperature. TEER values are expressed as percentages relative to the initial measurement (T0), which was set to 100%.

### 2.7. Paracellular Permeability Assay

After completing the protocol described above, the medium in the apical compartment was replaced with a 0.1 mM fluorescein isothiocyanate (FITC) solution, and cells were incubated at 37 °C for 30 min. Subsequently, 200 μL of medium was collected from the basolateral chamber, and fluorescein permeation was monitored using a VICTOR™ X3 multilabel plate reader (PerkinElmer) with excitation at 480 nm and emission at 530 nm [25].

### 2.8. Immunofluorescence

Caco-2 cells were plated onto glass coverslips in 24-wells plates and cultured for five days until a confluent monolayer formed. Cells were subsequently treated for 24 h with increasing concentrations of Spinetoram and Spirotetramat (0.5, 5, and 50 μM). After treatment, cells were washed, fixed with 4% paraformaldehyde, and permeabilized with PBS containing 0.1% Triton X-100. Immunostaining was performed by incubating cells with a mouse anti-Occludin primary antibody for 1 h at 37 °C. Samples were then incubated for 1 h at 37 °C with a FITC-conjugated Alexa Fluor 488 secondary antibody. Nuclear counterstaining was performed with Hoechst at room temperature. Coverslips were mounted on glass slides using Fluoromount-G™ Mounting Medium (Invitrogen, Carlsbad, CA, USA), and images were acquired with a LSM 800 confocal microscope (60× objective) with ZEN 2.1 Blue Edition software (Carl Zeiss, Jena, Germany).

### 2.9. Data Analysis

Results are reported as mean ± standard deviation (SD) or standard error of the mean (SEM). Statistical differences between groups were assessed using one-way or two-way analysis of variance (ANOVA), followed by Tukey’s multiple comparisons test. Statistical analyses were performed using GraphPad Prism 10 (GraphPad Software, San Diego, CA, USA). Differences were considered statistically significant at *p*-values below 0.05 (**** *p* ≤ 0.0001, *** *p* ≤ 0.001, ** *p* ≤ 0.01, * *p* ≤ 0.05, compared with control). All the experiments were performed at least three times (*n* = 3; biological replicates).

## 3. Results

### 3.1. Insecticides Promote a Pro-Inflammatory Macrophage Phenotype

**Figure 1:**
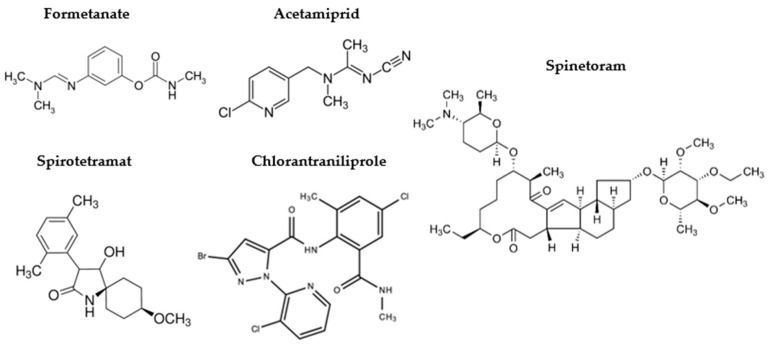
Chemical structures of the insecticides Formetanate, Acetamiprid, Spirotetramat, Chlorantraniliprole and Spinetoram.

A panel of insecticides widely used in agricultural practices, particularly in crops such as tomato and table grapes, was supplied by ReAgri S.r.l. (Figure 1) and evaluated for their immunomodulatory activity in PBMC-derived human macrophages. These low-molecular-weight compounds were selected based on their moderate-to-high frequency of agricultural application and their propensity to leave medium-to-high environmental residues.

**Figure 2:**
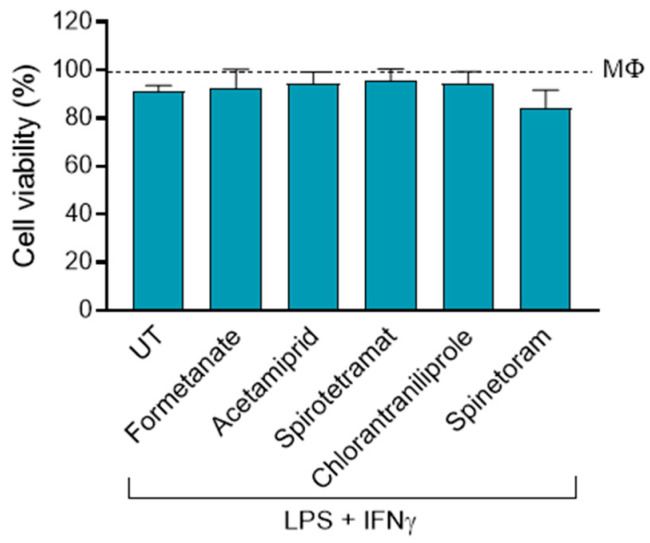
Cell viability of M1-like macrophages (differentiated with LPS+IFNγ) treated or not treated (UT) with 50 µM of the indicated insecticide. Data (mean ± SEM; *n* = 3) are expressed as % considering control macrophages not activated with LPS+IFNγ (MΦ) as 100%.

Considering that previous studies have shown that exposure to insecticides can induce inflammatory responses in both in-vivo and in-vitro models [15], we investigated whether these compounds could affect macrophage viability and polarization. To address this, primary human macrophages derived from PBMCs were polarized toward an M1-like phenotype using LPS and IFNγ, in the presence or absence of the insecticides Formetanate, Acetamiprid, Spirotetramat, Spinetoram, and Chlorantraniliprole. Cell viability and the expression of key M1-associated markers were subsequently analyzed. Notably, insecticide treatment did not affect the viability of M1 macrophages, even at the highest concentration tested (Figure 2).

**Figure 3:**
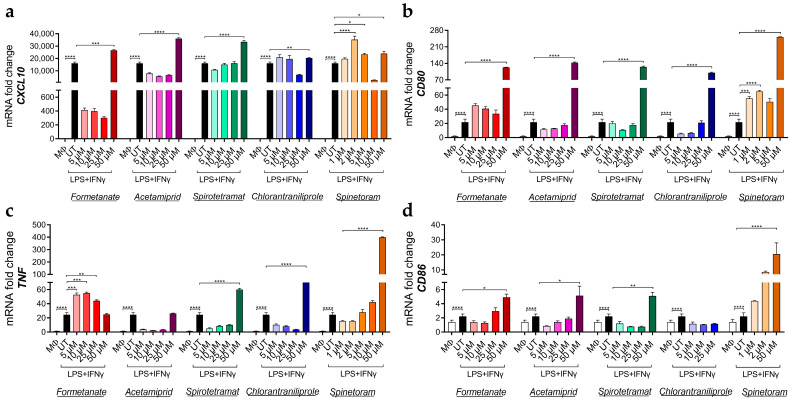
mRNA expression levels of *CXCL10* (**a**), *CD80* (**b**), *TNF* (**c**), and *CD86* (**d**) in M1-like macrophages treated (colored bars) or not treated (UT, black bars) with different concentrations of the indicated insecticides. Data (mean ± SEM; *n* = 3) are expressed as fold change compared with MΦ control macrophages (white bars). Statistical significances are calculated by using an ordinary one-way ANOVA test (* *p*  ≤  0.05; ** *p*  ≤  0.01; *** *p*  ≤  0.001; **** *p*  ≤  0.0001).

**Figure 4:**
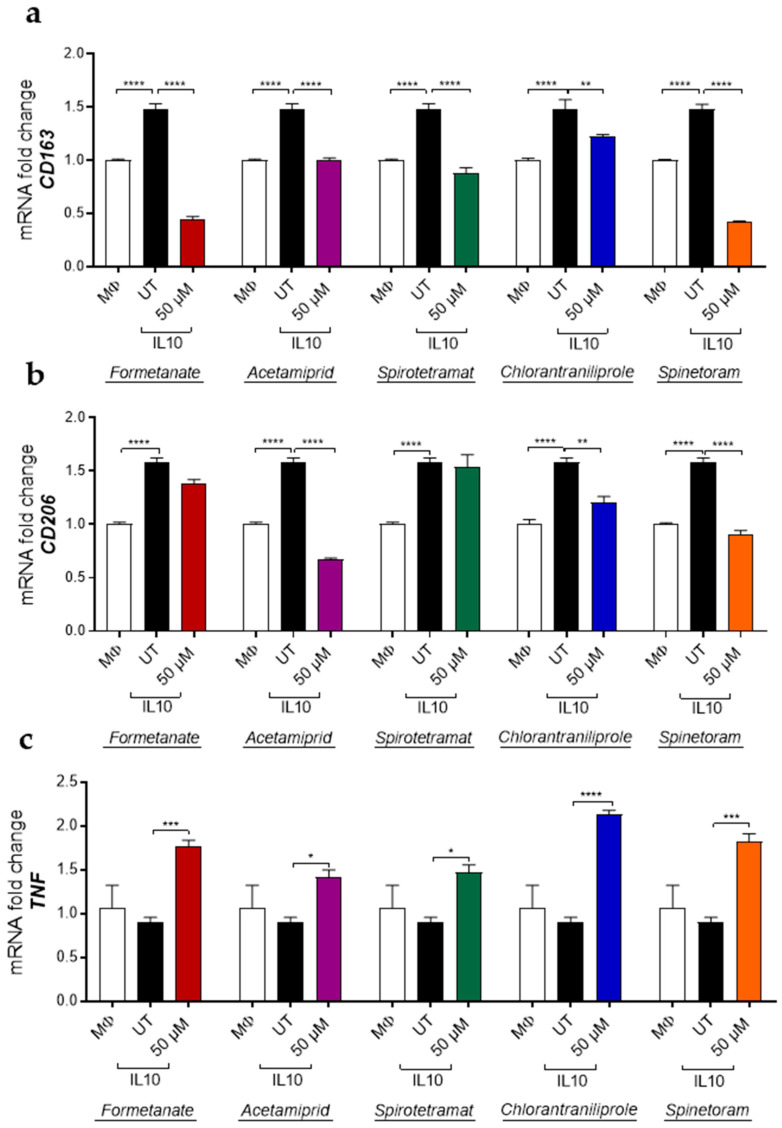
mRNA expression levels of *CD163* (**a**), *CD206* (**b**) and *TNF* (**c**) in M2-like macrophages treated (colored bars) or not treated (UT, black bars) with the indicated insecticides. Data (mean ± SEM; *n* = 3) are expressed as fold change compared with MΦ control macrophages (white bars). Statistical significances are calculated by using an ordinary one-way ANOVA test (* *p*  ≤  0.05; ** *p*  ≤  0.01; *** *p*  ≤  0.001; **** *p*  ≤  0.0001).

Concurrently, the expression of pro-inflammatory genes, including *CXCL10*, *CD80*, *TNF*, and *CD86*, was significantly increased by all compounds in a dose-dependent manner after 24 h of exposure (Figure 3a–d), indicating the enhancement of the pro-inflammatory macrophage phenotype. Furthermore, insecticide treatment of IL-10-polarized macrophages led to a significant downregulation in the expression of the M2-associated markers *CD163* and *CD206*, except for Formetanate and Spinetoram which decreased *CD163* only (Figure 4a,b).

**Figure 5:**
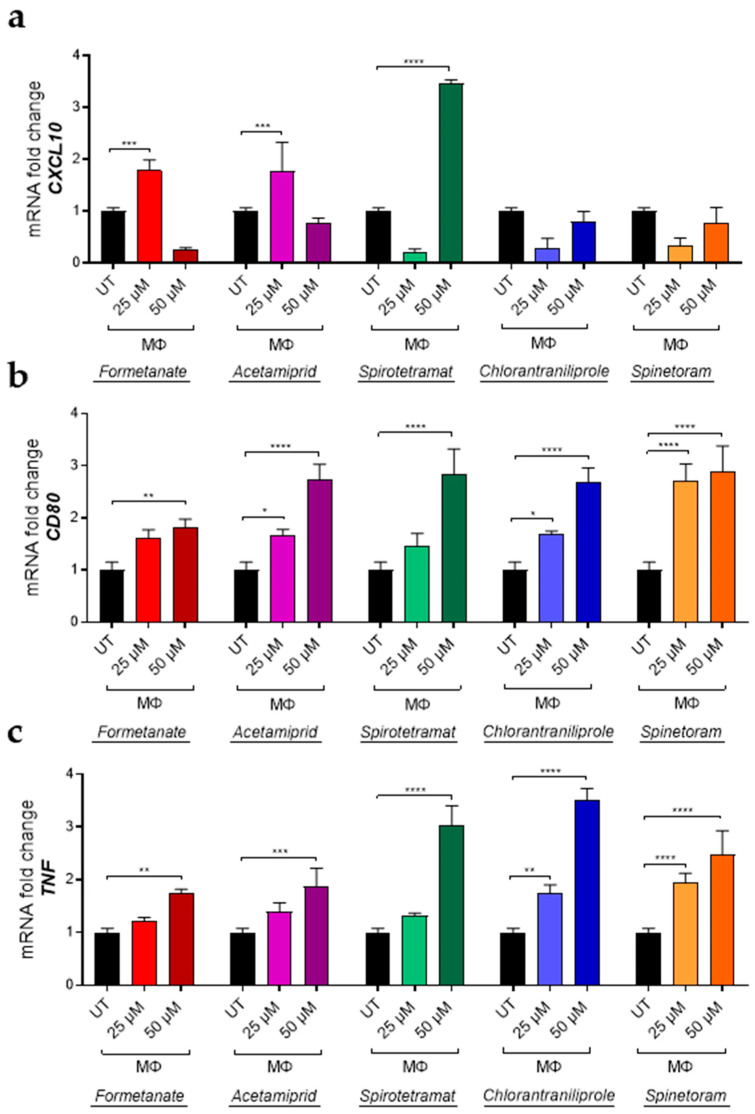
mRNA expression levels of *CXCL10* (**a**), *CD80* (**b**) and *TNF* (**c**) in MΦ macrophages treated (colored bars) or not treated (UT, black bars) with different concentrations of insecticides. Data (mean ± SD; *n* = 3) are expressed as fold change compared with UT. Statistical significances are calculated by using an ordinary one-way ANOVA test (* *p*  ≤  0.05; ** *p*  ≤  0.01; *** *p*  ≤  0.001; **** *p*  ≤  0.0001).

In parallel, insecticides also induced a marked increase in the expression of the M1-associated marker *TNF* compared with IL-10-stimulated macrophages (Figure 4c), indicating that these compounds can promote a shift from an anti-inflammatory M2-like phenotype toward a pro-inflammatory M1-like profile. To further investigate the impact of insecticides on macrophage polarization toward a pro-inflammatory phenotype, macrophages differentiated in the presence of granulocyte-macrophage colony-stimulating factor (GM-CSF; MΦ) were treated with insecticides using the experimental design described above. Under these conditions, insecticide treatment significantly increased the expression of M1-associated markers, including *CXCL10*, *CD80* and *TNF* (Figure 5a–c), further supporting the pronounced and broad pro-inflammatory effect induced by these compounds.

### 3.2. Insecticide Exposure Disrupts the Integrity of the Intestinal Epithelial Barrier

**Figure 6:**
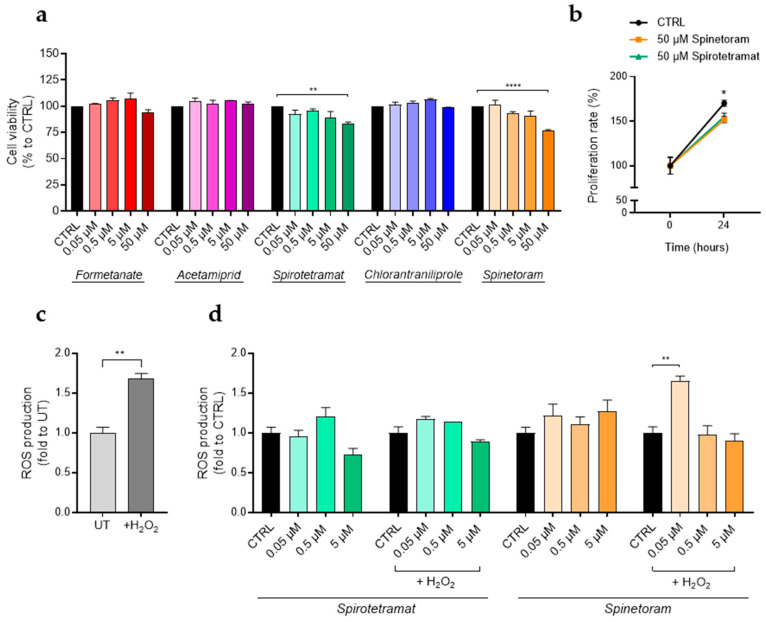
Impact of insecticides on cell viability and ROS production in Caco-2 cells. (**a**) Cell viability of Caco-2 cells treated with increasing concentrations of the indicated insecticide (colored bars). Data (mean ± SD; *n* = 3) are expressed as % considering untreated cells (CTRL, black bars) as 100%. (**b**) Proliferation rate of Caco-2 cells after 24 h of treatment with 50 µM of Spinetoram or Spirotetramat (green and orange lines). Proliferation of CTRL (black line) cells is reported and used as control. Cell counts (mean ± SD; *n* = 3) at 24 h were normalized to the initial cell number (0 h) and expressed as proliferation rate %. (**c**) ROS production levels in Caco-2 cells treated and not treated (UT) with H_2_O_2_. Data (mean ± SD; *n* = 3) are expressed as fold change compared with UT. (**d**) ROS production levels in Caco-2 cells treated with increasing concentrations of the indicated insecticide (green and orange bars), in basal condition and oxidative stress condition induced with H_2_O_2_. Data (mean ± SD; *n* = 3) are expressed as fold change compared with untreated cells (CTRL, black bars). Statistical significance is calculated by using an ordinary one-way ANOVA test (* *p*  ≤  0.05; ** *p*  ≤  0.01; **** *p*  ≤  0.0001).

Before reaching the liver and other organs, insecticides must first cross the intestinal epithelium. The homeostasis of this epithelial barrier is continuously challenged by external insults and depends on a finely regulated interplay among immune responses, epithelial components, and a balanced microbiota [26]. In this context, the human colorectal adenocarcinoma cell line Caco-2 was used as an in-vitro model of the intestinal epithelium to further evaluate the possible detrimental effects of insecticide exposure on epithelial barrier integrity [27]. We first examined whether treatments with insecticides for 24 h at concentrations ranging from 0.05 to 50 μM affected Caco-2 cell viability (Figure 6a).

No effects on cell viability were observed after treatment with the insecticides tested, except for Spirotetramat and Spinetoram, which, at the highest concentration, displayed approximately a 20% decrease in cell viability (Figure 6a). To assess whether this reduction was attributable to cell death or decreased cell growth, proliferation assays on Caco-2 cells, treated or not treated with 50 µM of Spinetoram or Spirotetramat for 24 h, were performed. Growth curves obtained, normalized to cell numbers at 0 h (Figure 6b), indicate that the observed decreased viability with 50 µM of Spinetoram and Spirotetramat treatments reflects a reduction in cell proliferation and not cell death. It can therefore be concluded that the two molecules exert a cytostatic rather than a cytotoxic effect at a concentration of 50 µM. The impact of Spirotetramat and Spinetoram on ROS production was subsequently evaluated under both basal and H_2_O_2_-induced oxidative stress conditions (Figure 6c). Results showed that Spirotetramat had no significant effect on ROS production under either basal or oxidative stress conditions, whereas 0.05 µM of Spinetoram significantly enhanced ROS production in cells exposed to oxidative stress (Figure 6d).

**Figure 7:**
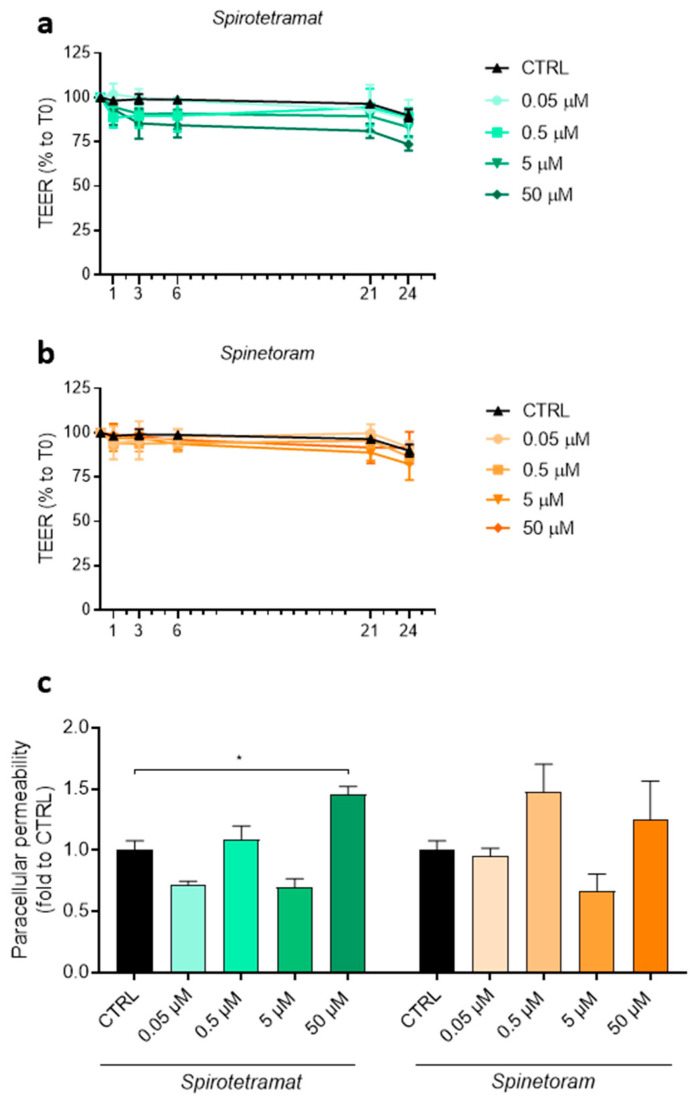
Effects of Spirotetramat and Spinetoram on TEER and paracellular permeability in Caco-2 cells. (**a**,**b**) TEER of Caco-2 cells treated (colored lines) or not treated (CTRL, black lines) with different concentrations of Spirotetramat (**a**) and Spinetoram (**b**). Data (mean ± SD; *n* = 3) are expressed as % related to time 0 h (T0). Statistical significance is calculated by using an ordinary two-way ANOVA test. (**c**) Paracellular permeability of Caco-2 cells treated with different concentrations of Spirotetramat and Spinetoram (green and orange bars). Data (mean ± SD; *n* = 3) are expressed as fold change compared with untreated (CTRL, black bars) cells. Statistical significance is calculated by using an ordinary one-way ANOVA test (* *p*  ≤  0.05).

To further assess the impact of these insecticides on intestinal barrier function, transepithelial electrical resistance (TEER) was monitored over a period of 24 h following insecticide exposure. Under these conditions, both Spirotetramat and Spinetoram did not induce significant changes in TEER values (Figure 7a,b). However, paracellular permeability assays revealed increased permeability following treatment with both compounds, reaching statistical significance with 50 μM Spirotetramat (Figure 7c), indicating epithelial barrier disruption.

**Figure 8:**
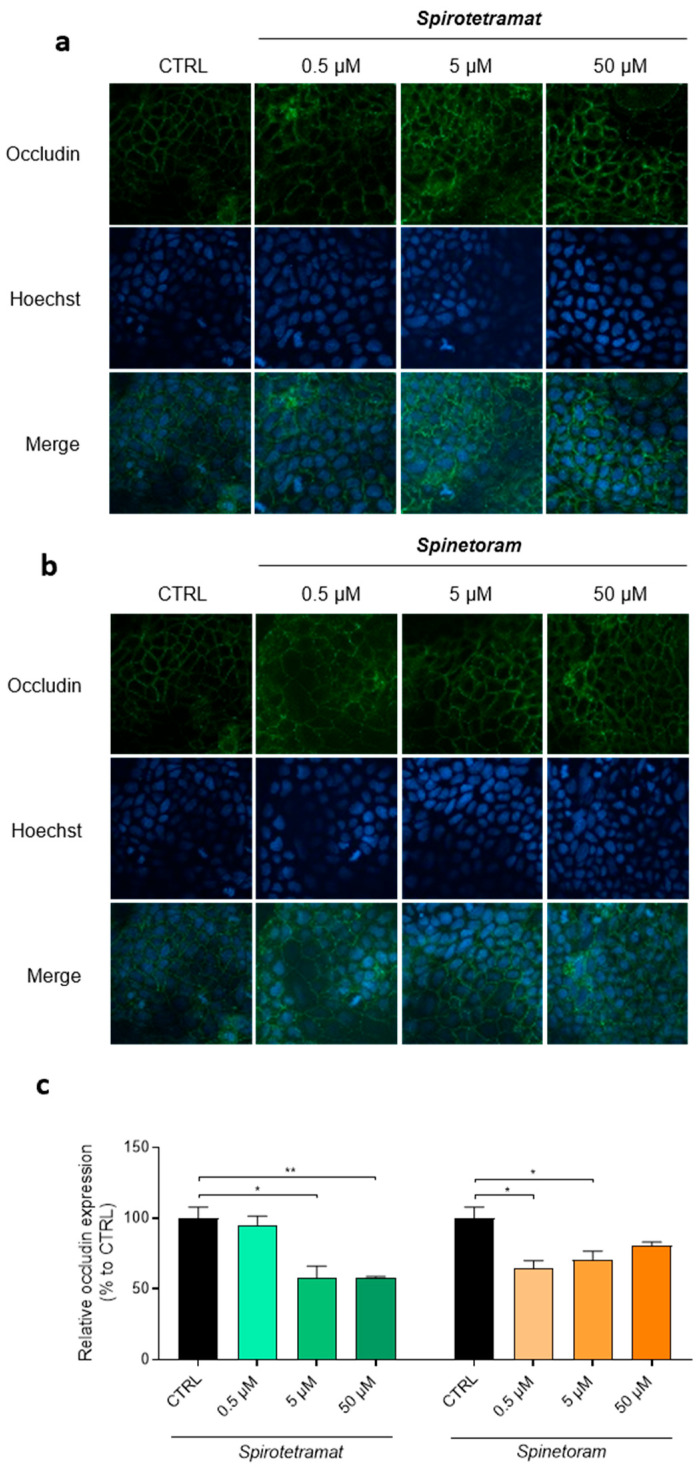
Distribution and expression of occludin in Caco-2 cells treated with Spirotetramat and Spinetoram. (**a**,**b**) Immunofluorescence of occludin and nuclei in Caco-2 cells treated or not treated (CTRL) with different concentrations of Spirotetramat (**a**) and Spinetoram (**b**). Representative images of three biological replicates are reported. (**c**) Relative expression levels of occludin in Caco-2 cells treated with increasing concentrations of Spirotetramat and Spinetoram (green and orange bars). Data (mean ± SD; *n* = 3) are expressed as % considering untreated cells (CTRL, black bars) as 100%. Statistical significance is calculated by using an ordinary one-way ANOVA test (* *p*  ≤  0.05; ** *p*  ≤  0.01).

The observed intestinal barrier alterations were further corroborated by analyzing the distribution and expression of occludin, a key tight junction protein essential for maintaining epithelial integrity and barrier function [28]. Coherently, exposure to both Spinetoram and Spirotetramat resulted in altered occludin localization, which appeared progressively less organized with increasing compound concentrations (Figure 8a,b). Moreover, a significant reduction in occludin expression was observed following treatment (Figure 8c) with both compounds, indicating the impairment of tight junction integrity.

Taken together, these findings demonstrate that the insecticides Spirotetramat and Spinetoram, particularly at higher concentrations, impair intestinal epithelial barrier integrity through mechanisms involving occludin abnormalities, leading to increased paracellular permeability.

## 4. Discussion

In this study, we demonstrate that a panel of widely used agricultural insecticides exerts a significant immunomodulatory impact on human macrophages and disrupts intestinal epithelial barrier integrity in-vitro. These findings provide mechanistic insights into how exposure to insecticide residues may contribute to inflammatory dysregulation and intestinal epithelial barrier dysfunction in humans.

As reported in Section 2, the insecticides were tested at concentrations that fall within the maximum residue limits commonly reported in fruits and vegetables. Therefore, the concentrations used in this study can be considered relevant for evaluating their potential biological effects on macrophages and on the gut epithelial barrier. Furthermore, the selected concentration range is comparable to that employed in previous in-vitro studies using different human cell culture models to investigate the cellular and molecular effects induced by insecticide exposure [29,30,31,32]. Taken together, these considerations support the toxicological relevance of the experimental conditions adopted in this study and strengthen the interpretation of the observed effects on macrophages and the epithelial intestinal barrier.

All tested insecticides induced a pronounced polarization toward a pro-inflammatory phenotype in human macrophages, as demonstrated by the dose-dependent up-regulation of canonical M1 markers, *CXCL10*, *CD80*, *CD86*, and *TNF*. Importantly, these effects were observed in the absence of detectable cytotoxicity, suggesting that insecticides act as direct functional modulators of immune response rather than non-specific toxicants. This observation is consistent with previous studies showing that pesticide exposure can activate inflammation-related signaling pathways, such as NF-κB and MAPK, without compromising cellular viability [15,16]. Moreover, similar immune-activating properties have been demonstrated for neonicotinoids and carbamates, two major classes of insecticides, which stimulate cytokine production and promote the expression of pro-inflammatory genes in both human and murine immune cells [17,33,34].

Our data indicate that treatment with insecticides significantly downregulates M2-associated markers (*CD163* and *CD206*) in IL-10 polarized macrophages, while concomitantly inducing the expression of *TNF*. These data depict an immune scenario characterized by macrophage reprogramming toward a pro-inflammatory phenotype, even under anti-inflammatory conditions. Such interference with macrophage plasticity may have important implications for immune homeostasis, as the balance between M1 and M2 phenotypes is critical for physiological processes such as inflammation resolution, tissue remodeling and immune tolerance [13,35]. Persistent skewing toward an M1-like state has been linked to autoimmune pathologies and chronic inflammatory diseases [14,36,37], raising serious concerns about the long-term immunological consequences of insecticide exposure.

Beyond immune modulation, our data revealed that Spirotetramat and Spinetoram impair intestinal epithelial barrier function, with Spirotetramat exerting the most pronounced effects. Increased paracellular permeability demonstrated a weakening of epithelial integrity, accompanied by altered localization and reduced expression of occludin, a key tight junction protein. Tight junction disruption is a recognized mechanism underlying increased intestinal permeability and systemic inflammation [28]. Furthermore, potential perturbations in fatty acid synthesis resulting from the inhibition of acetyl-CoA carboxylase by Spirotetramat may alter lipid homeostasis and cytoskeletal dynamics, processes that are essential for the stability of tight junctions [38,39]. Such alterations could promote the redistribution or degradation of junction proteins, including occludin, ultimately leading to increased paracellular permeability, as observed in the present study. Consistent with our findings, several environmental contaminants, including pesticides and foodborne xenobiotics, have been reported to destabilize tight junction complexes and promote epithelial leakiness [26,27,40].

Generally, our results indicate that barrier integrity impairment induced by Spinetoram or Spirotetramat is not associated with oxidative stress, as no significant ROS production was detected, even in the presence of H_2_O_2_ (Figure 6d). This suggests that ROS are effectively neutralized by endogenous antioxidant defenses. Non-oxidative mechanisms, such as cytoskeletal remodeling, membrane perturbation or direct interference with the turnover of junctional proteins [41,42,43,44], may underlie epithelial damage. Indeed, ROS-independent alterations of tight junctions have been reported for other xenobiotics affecting epithelial homeostasis [10]. Notably, Spinetoram treatment induced an increase in ROS only at a 0.05 µM concentration, a level at which no paracellular permeability or occludin abnormalities were observed. This implies that ROS production remains well-compensated across most tested Spinetoram concentrations; however, 0.05 µM may be insufficient to trigger a rapid, adaptive antioxidant response.

Collectively, these findings support a model in which insecticides act at multiple biological levels: promoting inflammatory macrophage polarization while simultaneously weakening the intestinal barrier. Because the gut epithelium represents a primary route of exposure, barrier disruption may facilitate increased systemic absorption of insecticides, thereby amplifying immune activation and systemic inflammatory responses. This epithelial-immune axis has emerged as a central mechanism linking environmental exposures to chronic inflammation, metabolic dysfunction, and autoimmune diseases [11].

Despite the strengths of this study, some limitations should be acknowledged. These include the use of acute exposure paradigms and in-vitro models that do not fully recapitulate metabolic transformation, microbiota interactions, or chronic exposure scenarios. Future studies, employing long-term exposure models, that consider the metabolic transformation of insecticides, interactions with the gut microbiota, immune cell co-culture systems and in-vivo validation, will be crucial to better assess the translational relevance of our findings and to delineate the molecular pathways underlying insecticide-driven immunotoxicity. Furthermore, our study was focused on the specific effects of individual compounds on macrophage responses and gut epithelial barrier integrity. However, in real exposure scenarios, these insecticides are likely to occur in combination with other pesticides. Therefore, future studies will investigate the potential impact of pesticide mixtures on macrophage activation and on the integrity and function of the intestinal epithelial barrier.

## 5. Conclusions

Overall, this study demonstrates that commonly used agricultural insecticides induce pro-inflammatory responses and intestinal barrier disruption in human in-vitro models. These compounds promote macrophage polarization toward an M1-like phenotype, suppress anti-inflammatory markers, and jeopardize intestinal epithelial integrity by disrupting tight junction architecture.

Our findings support concerns that long-term, low-level exposure to pesticide residues may contribute to immune dysregulation, increased intestinal permeability, and inflammation-related diseases. These results highlight the need for more comprehensive toxicological evaluation frameworks that incorporate immunological and epithelial barrier-related endpoints, in addition to traditional cytotoxicity assessments. Finally, this work supports the consideration of certain insecticides as potential environmental immunotoxins and underscores the importance of developing safer and more sustainable pest-control strategies.

## Data Availability

The original contributions presented in this study are included in the article. Further inquiries can be directed to the corresponding authors.

## Abbreviations

The following abbreviations are used in this manuscript:

PBMCs	Peripheral Blood Mononuclear Cells
GM-CSF	Granulocyte-Macrophage Colony-Stimulating Factor
M-CSF	Macrophage Colony-Stimulating Factor
LPS	Lipopolysaccharide
IFNγ	Interferon gamma
H_2_-DCF-DA	2′,7′-Dichlorodihydrofluorescein diacetate
ROS	Reactive Oxygen Species
IL-10	Interleukin-10
TEER	Transepithelial Electrical Resistance
NF-κB	Nuclear Factor kappa-light-chain-enhancer of activated B cells
MAPK	Mitogen-Activated Protein Kinase
